# Supplementary material for: A Serodiagnostic IgM ELISA to Detect Acute Cytauxzoonosis
Source: Pathogens. 2022 Oct 14;11(10):1183. doi: 10.3390/pathogens11101183 (PMC9611129; doi:10.3390/pathogens11101183)
Supplement: Supplementary file 1 [file pathogens-11-01183-s001.zip › Kao et al 2022 Supplemental Tables.pdf]

Table S1.

Result of Blood Smear Evaluation† of Group 2 Blood Samples.

Cat ID	IgM ELISA result (percent positive)	Total number of piroplasms observed	Total number of schizont-laden monocytes in 2 blood smears	Determination of *C. felis* infection (ddPCR)
201	104.2	344	0	Positive
202	20.6	0	0	Positive
204	34.1	61	4	Positive
205	9.4*	2	0	Positive
206	74.7	2	0	Positive
207	107.5	33	0	Positive
208	59.4	1	0	Positive
209	8.9*	1	0	Positive
210	40.8	14	1	Positive
211	63.4	3	0	Positive
212	28.4	208	0	Positive
213	52.2	11	0	Positive
214	104.7	11	0	Positive
215	109.6	542	0	Positive
216	102.9	10	11	Positive
217	75.4	2	0	Positive
218	115.0	8	0	Positive
219	112.2	7	0	Positive
220	98.6	28	0	Positive
221	110.0	10	2	Positive
222	98.8	N/A	N/A	Positive
223	88.8	7	0	Positive
224	70.8	21	2	Positive
225	80.7	21	0	Positive
226	78.9	52	0	Positive
227	69.9	5	0	Positive
228	43.2	460	4	Positive
229	88.3	203	0	Positive
230	88.5	179	1	Positive
231	100.0	183	0	Positive
232	64.2	9	0	Positive
233	50.1	7	0	Positive
234	55.6	0	2	Positive
235	55.9	608	0	Positive
236	22.8	N/A	N/A	Positive
237	50.6	164	0	Positive

*Weak positive IgM ELISA result

† For each sample, duplicate blood smears were made when available and evaluated for presence of *C. felis* organisms. The method of blood smear evaluation was described as previously published (Kao et al., 2021). Among the 36 samples, blood smears were not available in 2 samples (222, 236) and were marked as N/A (not applicable).

Table S2.

Result of Blood Smear Evaluation[†] of Group 3 Blood Samples.

Cat ID	IgM ELISA result (percent positive)	Total number of piroplasms observed	Total number of schizont-laden monocytes in 2 blood smears	Determination of *C. felis* infection (ddPCR)
301	5.4	0	0	Negative
302	8.3	0	0	Negative
303	4.1	0	0	Negative
304	12.3*	0	0	Negative
305	5.6	0	0	Negative
306	8.2	0	0	Negative
307	4.5	0	0	Negative
308	10.0*	0	0	Negative
309	3.2	0	0	Negative
310	3.8	0	0	Negative

*Weak positive IgM ELISA result

[†] For each sample, duplicate blood smears were made when available and evaluated for presence of *C. felis* organisms. The method of blood smear evaluation was described as previously published (Kao et al., 2021).
