# Supplementary material for: A Serodiagnostic IgM ELISA to Detect Acute Cytauxzoonosis
Source: Pathogens. 2022 Oct 14;11(10):1183. doi: 10.3390/pathogens11101183 (PMC9611129; doi:10.3390/pathogens11101183)
Supplement: Supplementary file 1 [file pathogens-11-01183-s001.zip › Kao et al ELISA data.pdf]

Data Summary

Group 1

Cat ID	IgM ELISA result (percent positive)	IgG ELISA result (percent positive)	*C. felis* cox3 ddPCR result
101	5.9	5.2	Negative
102	3.9	4.9	Negative
103	3.7	4.8	Negative
104	5.7	6.6	Negative
105	5.2	6.3	Negative
106	4.6	5.8	Negative
107	4.9	8.7	Negative
108	4.2	6	Negative
109	19.1	6.4	Negative
110	5.1	4.3	Negative
111	4.7	5.2	Negative
112	4.3	5	Negative
113	10.2	4.2	Negative
114	3.4	5.5	Negative
115	6.7	6.2	Negative
116	4.1	5.2	Negative
117	4.3	5.7	Negative
118	6.2	5.1	Negative
119	7	7.1	Negative
120	6.8	6.8	Negative
121	4	6.7	Negative
122	7.3	6.9	Negative

Group 2

Cat ID	IgM ELISA result (percent positive)	IgG ELISA result (percent positive)	*C. felis* cox3 ddPCR result
201	104.2	43.5	Positive
202	20.6	15.3	Positive
204	34.1	10.8	Positive
205	9.4	53.6	Positive
206	74.7	18.7	Positive
207	107.5	105.1	Positive
208	59.4	35.5	Positive
209	8.9	45.5	Positive
210	40.8	38.6	Positive
211	63.4	11.1	Positive
212	28.4	32.7	Positive
213	52.2	58.1	Positive
214	104.7	21.6	Positive
215	109.6	56.5	Positive
216	102.9	78.7	Positive
217	75.4	12.6	Positive
218	115	79.7	Positive
219	112.2	92.9	Positive
220	98.6	54.2	Positive
221	110	79.7	Positive
222	98.8	79.3	Positive
223	88.8	76.1	Positive
224	70.8	49.4	Positive
225	80.7	31.6	Positive
226	78.9	71.5	Positive
227	69.9	81.7	Positive
228	43.2	53.6	Positive
229	88.3	74.5	Positive
230	88.5	36.5	Positive
231	100	71.4	Positive
232	64.2	28.8	Positive
233	50.1	28.1	Positive
234	55.6	50.4	Positive
235	55.9	12.5	Positive
236	22.8	79.7	Positive
237	50.6	14.3	Positive

Group 3

Cat ID	IgM ELISA result (percent positive)	IgG ELISA result (percent positive)	*C. felis* cox3 ddPCR result
301	5.4	5.2	Negative
302	8.3	3.7	Negative
303	4.1	4.1	Negative
304	12.3	3.4	Negative
305	5.6	5.4	Negative
306	8.2	4.3	Negative
307	4.5	4.2	Negative
308	10	2.6	Negative
309	3.2	2.6	Negative
310	3.8	48.3	Negative

Group 4

Cat ID	IgM ELISA result (percent positive)	IgG ELISA result (percent positive)	*C. felis* cox3 ddPCR result
401	7.5	15.6	Positive
402	17.3	15.9	Positive
403	40.3	30.3	Positive
404	11.4	54.6	Positive

IgM ELISA

Strong postitive cut-off: 19.85 percent positive

Weak positive cut-off: 8.60 percent positive

IgG ELISA

Positive cut-off: 9.90 percent positive

IgM Raw data

Original ID	Cat ID	ddPCR	Plate I			Plate II			Plate I Mean	Plate I Std	Plate I CV	%PP	Plate II Mear	Plate II Std	Plate II CV	%PP	Inter plate m	Inter plate St	Interpate CV	All sample PP
Dexter	201	positive	3.0298	3.0019	5/6/21	3.431	3.325	5/6/21	3.01585	0.01972828	1%	98.7%	3.378	0.07495332	2%	109.6%	3.196925	0.25607872	8%	104.2%
0501 d0	202	positive	0.5047	0.5342	5/6/21	0.7667	0.7231	5/6/21	0.51945	0.02085965	4%	17.0%	0.7449	0.03082986	4%	24.2%	0.632175	0.15941722	25%	20.6%
0801 d0	204	positive	0.8787	0.9023	5/6/21	1.2015	1.1967	5/6/21	0.8905	0.01668772	2%	29.2%	1.1991	0.00339411	0%	38.9%	1.0448	0.21821315	21%	34.1%
1501 d0	205	positive	0.2525	0.2606	5/6/21	0.3314	0.3036	5/6/21	0.25655	0.00572756	2%	8.4%	0.3175	0.01965757	6%	10.3%	0.287025	0.04309816	15%	9.4%
1502 d0	206	positive	2.1745	2.0449	5/6/21	2.519	2.4219	5/6/21	2.1097	0.09164104	4%	69.1%	2.47045	0.06866007	3%	80.2%	2.290075	0.25508877	11%	74.7%
1503 d0	207	positive	3.271	3.2298	5/6/21	3.3502	3.3348	5/6/21	3.2504	0.0291328	1%	106.4%	3.3425	0.01088944	0%	108.5%	3.29645	0.06512453	2%	107.5%
1505 d0	208	positive	1.7005	1.6161	5/6/21	1.9838	1.9894	5/6/21	1.6583	0.05967981	4%	54.3%	1.9866	0.0039598	0%	64.5%	1.82245	0.23214316	13%	59.4%
1506 d0	209	positive	0.2431	0.2481	5/6/21	0.2981	0.3024	5/6/21	0.2456	0.00353553	1%	8.0%	0.30025	0.00304056	1%	9.7%	0.272925	0.03864339	14%	8.9%
1507 d0	210	positive	1.1209	1.1712	5/6/21	1.3311	1.381	5/6/21	1.14605	0.03556747	3%	37.5%	1.35605	0.03528463	3%	44.0%	1.25105	0.14849242	12%	40.8%
1508 d0	211	positive	1.7495	1.835	5/6/21	2.1052	2.0893	5/6/21	1.79225	0.06045763	3%	58.7%	2.09725	0.011243	1%	68.1%	1.94475	0.21566757	11%	63.4%
0901 d0	212	positive	0.7568	0.7898	5/6/21	0.9348	1.0039	5/6/21	0.7733	0.02333452	3%	25.3%	0.96935	0.04886108	5%	31.5%	0.871325	0.13862828	16%	28.4%
0902 d0	213	positive	1.6809	1.426	5/6/21	1.6444	1.6601	5/6/21	1.55345	0.18024152	12%	50.9%	1.65225	0.01110158	1%	53.6%	1.60285	0.06986215	4%	52.2%
Squeaky	214	positive	3.1672	3.1515	5/6/21	3.2647	3.2588	5/6/21	3.15935	0.01110158	0%	103.4%	3.26175	0.00417193	0%	105.9%	3.21055	0.07240773	2%	104.7%
0302 d0	215	positive	3.3665	3.3422	5/6/21	3.3821	3.3538	5/6/21	3.35435	0.01718269	1%	109.8%	3.36795	0.02001112	1%	109.3%	3.36115	0.00961665	0%	109.6%
0703 d0	216	positive	3.1092	3.0758	5/6/21	3.2328	3.2142	5/6/21	3.0925	0.02361737	1%	101.3%	3.2235	0.01315219	0%	104.6%	3.158	0.09263099	3%	102.9%
0903 d0	217	positive	2.1725	2.2089	5/6/21	2.4409	2.4353	5/6/21	2.1907	0.02573869	1%	71.7%	2.4381	0.0039598	0%	79.1%	2.3144	0.17493822	8%	75.4%
1201 d0	218	positive	3.528	3.5064	5/6/21	3.5286	3.5495	5/6/21	3.5172	0.01527351	0%	115.2%	3.53905	0.01477853	0%	114.9%	3.528125	0.01545028	0%	115.0%
1510 d0	219	positive	3.4321	3.4287	5/6/21	3.4203	3.4897	5/6/21	3.4304	0.00240416	0%	112.3%	3.455	0.04907321	1%	112.1%	3.4427	0.01739483	1%	112.2%
1511 d0	220	positive	2.951	2.9795	5/6/21	3.0901	3.0837	5/6/21	2.96525	0.02015254	1%	97.1%	3.0869	0.00452548	0%	100.2%	3.026075	0.08601954	3%	98.6%
0704 d0	221	positive	3.3562	3.4037	5/6/21	3.4236	3.3086	5/6/21	3.37995	0.03358757	1%	110.7%	3.3661	0.08131728	2%	109.2%	3.373025	0.00979343	0%	110.0%
0303 d0	222	positive	3.0256	2.9988	5/6/21	3.0646	3.0312	5/6/21	3.0122	0.01895046	1%	98.6%	3.0479	0.02361737	1%	98.9%	3.03005	0.02524371	1%	98.8%
1512 d0	223	positive	2.6525	2.667	5/6/21	2.8041	2.7747	5/6/21	2.65975	0.01025305	0%	87.1%	2.7894	0.02078894	1%	90.5%	2.724575	0.09167639	3%	88.8%
Pos Control			3.0711	3.0372	5/6/21	3.093	3.0696	5/6/21	3.05415	0.02397092	1%	100.0%	3.0813	0.0165463	1%	100.0%	3.067725	0.01919795	1%	100.0%
Neg Control			0.2499	0.2418	5/6/21	0.2909	0.287	5/6/21	0.24585	0.00572756	2%	8.0%	0.28895	0.00275772	1%	9.4%	0.2674	0.0304763	11%	8.7%
0304 d0	224	positive	2.6429	2.6499	5/4/21	3.0537	2.9874	5/4/21	2.6464	0.00494975	0%	66.2%	3.02055	0.04688118	2%	75.5%	2.833475	0.264564	9%	70.8%
0305 d0	225	positive	3.1303	3.1141	5/4/21	3.3377	3.3361	5/4/21	3.1222	0.01145513	0%	78.1%	3.3369	0.00113137	0%	83.4%	3.22955	0.15181583	5%	80.7%
1202 d0	226	positive	3.14	3.1269	5/4/21	3.0042	3.3562	5/4/21	3.13345	0.0092631	0%	78.3%	3.1802	0.24890159	8%	79.5%	3.156825	0.03305724	1%	78.9%
1203 d0	227	positive	2.5585	2.5867	5/4/21	3.0293	3.012	5/4/21	2.5726	0.01994041	1%	64.3%	3.02065	0.01223295	0%	75.5%	2.796625	0.31681919	11%	69.9%
Sac Stray	228	positive	1.6439	1.6369	5/4/21	1.8279	1.7965	5/4/21	1.6404	0.00494975	0%	41.0%	1.8122	0.02220315	1%	45.3%	1.7263	0.12148095	7%	43.2%
Potty Mouth	229	positive	3.5434	3.526	5/4/21	3.5749	3.487	5/4/21	3.5347	0.01230366	0%	88.4%	3.53095	0.06215469	2%	88.3%	3.532825	0.00265165	0%	88.3%
1002	230	positive	3.5215	3.4915	5/4/21	3.5715	3.5774	5/4/21	3.5065	0.0212132	1%	87.7%	3.57445	0.00417193	0%	89.4%	3.540475	0.04804791	1%	88.5%
Bandit	231	positive	3.4054	3.4354	5/4/21	3.5008	3.4584	5/4/21	3.4204	0.0212132	1%	85.5%	3.4796	0.02998133	1%	87.0%	3.45	0.04186072	1%	86.3%
Little Guy	232	positive	2.3663	2.3183	5/4/21	2.8537	2.7393	5/4/21	2.3423	0.03394113	1%	58.6%	2.7965	0.08089302	3%	69.9%	2.5694	0.3211679	12%	64.2%
0701 d0	233	positive	1.833	1.824	5/4/21	2.2404	2.1109	5/4/21	1.8285	0.00636396	0%	45.7%	2.17565	0.09157033	4%	54.4%	2.002075	0.24547212	12%	50.1%
0601 d0	234	positive	2.0776	2.1724	5/4/21	2.3496	2.3031	5/4/21	2.125	0.06703372	3%	53.1%	2.32635	0.03288047	1%	58.2%	2.225675	0.14237595	6%	55.6%
1504-	301	negative	0.1973	0.1937	5/4/21	0.2459	0.2342	5/4/21	0.1955	0.00254558	1%	4.9%	0.24005	0.00827315	3%	6.0%	0.217775	0.03150161	14%	5.4%
0702-	302	negative	0.3035	0.2864	5/4/21	0.3705	0.3734	5/4/21	0.29495	0.01209153	4%	7.4%	0.37195	0.00205061	1%	9.3%	0.33345	0.05444722	16%	8.3%
1509-	303	negative	0.1489	0.1491	5/4/21	0.1771	0.1859	5/4/21	0.149	0.00014142	0%	3.7%	0.1815	0.00622254	3%	4.5%	0.16525	0.02298097	14%	4.1%
1001-	304	negative	0.4283	0.4352	5/4/21	0.5453	0.5554	5/4/21	0.43175	0.00487904	1%	10.8%	0.55035	0.00714178	1%	13.8%	0.49105	0.08386286	17%	12.3%
1513-	305	negative	0.1934	0.193	5/4/21	0.2561	0.2585	5/4/21	0.1932	0.00028284	0%	4.8%	0.2573	0.00169706	1%	6.4%	0.22525	0.04532554	20%	5.6%
Fat Tony-	306	negative	0.293	0.285	5/4/21	0.3563	0.3724	5/4/21	0.289	0.00565685	2%	7.2%	0.36435	0.01138442	3%	9.1%	0.326675	0.0532805	16%	8.2%
Squeak-	307	negative	0.162	0.1557	5/4/21	0.1977	0.2012	5/4/21	0.15885	0.00445477	3%	4.0%	0.19945	0.00247487	1%	5.0%	0.17915	0.02870854	16%	4.5%
Jamiraquoi-	308	negative	0.3572	0.367	5/4/21	0.4498	0.4324	5/4/21	0.3621	0.00692965	2%	9.1%	0.4411	0.01230366	3%	11.0%	0.4016	0.05586144	14%	10.0%
Prince-	309	negative	0.1127	0.1112	5/4/21	0.1466	0.1454	5/4/21	0.11195	0.00106066	1%	2.8%	0.146	0.00084853	1%	3.7%	0.128975	0.02407699	19%	3.2%
0306 d0-	310	negative	0.1374	0.1403	5/4/21	0.1641	0.1651	5/4/21	0.13885	0.00205061	1%	3.5%	0.1646	0.00070711	0%	4.1%	0.151725	0.018208	12%	3.8%
Pepper	235	positive	2.1448	2.1401	5/4/21	2.3425	2.3111	5/4/21	2.14245	0.0033234	0%	53.6%	2.3268	0.02220315	1%	58.2%	2.234625	0.13035514	6%	55.9%
Aggie	401	positive	0.253	0.2591	5/4/21	0.3459	0.3431	5/4/21	0.25605	0.00431335	2%	6.4%	0.3445	0.0019799	1%	8.6%	0.300275	0.06254359	21%	7.5%
BJ	402	positive	0.6292	0.6094	5/4/21	0.7453	0.777	5/4/21	0.6193	0.01400071	2%	15.5%	0.76115	0.02241528	3%	19.0%	0.690225	0.1003031	15%	17.3%
Goose	403	positive	1.3083	1.4817	5/4/21	1.7713	1.8809	5/4/21	1.395	0.12261232	9%	34.9%	1.8261	0.0774989	4%	45.7%	1.61055	0.30483373	19%	40.3%
AJ	404	positive	0.4024	0.404	5/4/21	0.509	0.5119	5/4/21	0.4032	0.00113137	0%	10.1%	0.51045	0.00205061	0%	12.8%	0.456825	0.0758372	17%	11.4%
Pos Control			4	4	5/4/21	4	4	5/4/21	4	0	0%	100.0%	4	0	0%	100.0%	4	0	0%	100.0%
Neg Control			0.2649	0.265	5/4/21	0.3166	0.3217	5/4/21	0.26495	7.0711E-05	0%	6.6%	0.31915	0.00360624	1%	8.0%	0.29205	0.03832519	13%	7.3%
95	101	negative	0.2355	0.2211	5/5/21	0.2031	0.2139	5/5/21	0.2283	0.01018234	4%	6.8%	0.2085	0.00763675	4%	5.2%	0.2184	0.01400071	6%	5.9%
109	102	negative	0.1621	0.142	5/5/21	0.1381	0.1323	5/5/21	0.15205	0.01421285	9%	4.5%	0.1352	0.00410122	3%	3.4%	0.143625	0.01191475	8%	3.9%
452	103	negative	0.1488	0.1355	5/5/21	0.1249	0.1383	5/5/21	0.14215	0.00940452	7%	4.2%	0.1316	0.00947523	7%	3.3%	0.136875	0.00745998	5%	3.7%
575	104	negative	0.2128	0.2183	5/5/21	0.2099	0.1996	5/5/21	0.21555	0.00388909	2%	6.4%	0.20475	0.0072832	4%	5.1%	0.21015	0.00763675	4%	5.7%
559	105	negative	0.1934	0.1897	5/5/21	0.1992	0.1877	5/5/21	0.19155	0.0026163	1%	5.7%	0.19345	0.00813173	4%	4.8%	0.1925	0.0013435	1%	5.2%
613	106	negative	0.1821	0.1534	5/5/21	0.1744	0.1724	5/5/21	0.16775	0.02029396	12%	5.0%	0.1734	0.00141421	1%	4.3%	0.170575	0.00399515	2%	4.6%
664	107	negative	0.1754	0.1807	5/5/21	0.1834	0.184	5/5/21	0.17805	0.00374767	2%	5.3%	0.1837	0.00042426	0%	4.6%	0.180875	0.00399515	2%	4.9%
800	108	negative	0.1603	0.1587	5/5/21	0.1455	0.1548	5/5/21	0.1595	0.00113137	1%	4.7%	0.15015	0.00657609	4%	3.8%	0.154825	0.00661145	4%	4.2%
842	109	negative	0.6393	0.5865	5/12/21	0.5818	0.6147	5/12/21	0.6129	0.03733524	6%	19.2%	0.59825	0.02326381	4%	16.2%	0.605575	0.01035911	2%	17.6%
907	110	negative	0.1941	0.1913	5/5/21	0.1893	0.1778	5/5/21	0.1927	0.0019799	1%	5.7%	0.18355	0.00813173	4%	4.6%	0.188125	0.00647003	3%	5.1%
923	111	negative	0.1827	0.1733	5/5/21	0.1635	0.1712	5/5/21	0.178	0.0066468	4%	5.3%	0.16735	0.00544472	3%	4.2%	0.172675	0.00753069	4%	4.7%
1024	112	negative	0.1633	0.1618	5/5/21	0.155	0.1498	5/5/21	0.16255	0.00106066	1%	4.8%	0.1524	0.00367696	2%	3.8%	0.157475	0.00717713	5%	4.3%
1130	113	negative	0.3848	0.38	5/5/21	0.3556	0.3762	5/5/21	0.3824	0.00339411	1%	11.4%	0.3659	0.0145664	4%	9.1%	0.37415	0.01166726	3%	10.2%
1440	114	negative	0.1337	0.1301	5/5/21	0.1146	0.1224	5/5/21	0.1319	0.00254558	2%	3.9%	0.1185	0.00551543	5%	3.0%	0.1252	0.00947523	8%	3.4%
1474	115	negative	0.2613	0.2475	5/5/21	0.2367	0.2375	5/5/21	0.2544	0.00975807	4%	7.6%	0.2371	0.00056569	0%	5.9%	0.24575	0.01223295	5%	6.7%
9491	116	negative	0.1516	0.1458	5/5/21	0.1501	0.1527	5/5/21	0.1487	0.00410122	3%	4.4%	0.1514	0.00183848	1%	3.8%	0.15005	0.00190919	1%	4.1%
9687	117	negative	0.1622	0.1716	5/5/21	0.1459	0.1541	5/5/21	0.1669	0.0066468	4%	5.0%	0.15	0.00579828	4%	3.8%	0.15845	0.0119501	8%	4.3%
9857	118	negative	0.2234	0.265	5/5/21	0.1957	0.225	5/5/21	0.2442	0.02941564	12%	7.3%	0.21035	0.02071823	10%	5.3%	0.227275	0.02393556	11%	6.2%
zag1 d0	119	negative	0.2534	0.2694	5/5/21	0.26	0.2465	5/5/21	0.2614	0.01131371	4%	7.8%	0.25325	0.00954594	4%	6.3%	0.257325	0.00576292	2%	7.0%
mdo6 do	120	negative	0.2475	0.2698	5/5/21	0.242	0.24	5/5/21	0.25865	0.01576848	6%	7.7%	0.241	0.00141421	1%	6.0%	0.249825	0.01248043	5%	6.8%
zae1 d0	121	negative	0.1511	0.1511	5/5/21	0.1538	0.1384	5/5/21	0.1511	0	0%	4.5%	0.1461	0.01088944	7%	3.7%	0.1486	0.00353553	2%	4.0%
meb3 d0	122	negative	0.2705	0.2701	5/5/21	0.2592	0.2696	5/5/21	0.2703	0.00028284	0%	8.0%	0.2644	0.00735391	3%	6.6%	0.26735	0.00417193	2%	7.3%
0307 d0	236	positive	0.828	0.8339	5/5/21	0.8379	0.8528	5/5/21	0.83095	0.00417193	1%	24.7%	0.84535	0.01053589	1%	21.1%	0.83815	0.01018234	1%	22.8%
Charm	237	positive	1.8569	1.9159	5/5/21	1.8648	1.8127	5/5/21	1.8864	0.0417193	2%	56.1%	1.83875	0.03684026	2%	46.0%	1.862575	0.03369364	2%	50.6%
Pos Control			3.2141	3.5099	5/5/21	4	4	5/5/21	3.362	0.20916219	6%	100.0%	4	0	0%	100.0%	3.681	0.45113413	12%	100.0%
Pos Control			3.1601	3.2283	5/12/21	3.6122	3.7836	5/12/21	3.1942	0.04822468	2%	100.0%	3.6979	0.1211981	3%	100.0%	3.44605	0.35616969	10%	100.0%

IgG Raw Data

Cat name	Cat ID	ddPCR	Plate I		Date	Plate II		Date	Plate I Mean	Plate I Std	Plate I CV	%PP	Plate II Mear	Plate II Std	Plate II CV	%PP	Inter plate m	Inter plate St	Interpate CV	All sample PP
Dexter	201	positive	0.9427	0.9338	5/3/21	1.3311	1.3227	5/3/21	0.93825	0.00629325	1%	38.0%	1.3269	0.0059397	0%	48.4%	1.132575	0.27481705	24%	43.5%
0501 d0	202	positive	0.3597	0.3642	5/6/21	0.388	0.3614	5/6/21	0.36195	0.00318198	1%	15.7%	0.3747	0.01880904	5%	14.9%	0.368325	0.00901561	2%	15.3%
0801 d0	204	positive	0.24	0.2366	5/6/21	0.2932	0.2711	5/6/21	0.2383	0.00240416	1%	10.3%	0.28215	0.01562706	6%	11.2%	0.260225	0.03100663	12%	10.8%
1501 d0	205	positive	1.4091	1.3876	5/3/21	1.4233	1.3625	5/3/21	1.39835	0.0152028	1%	56.7%	1.3929	0.04299209	3%	50.8%	1.395625	0.00385373	0%	53.6%
1502 d0	206	positive	0.4166	0.3994	5/6/21	0.4834	0.5041	5/6/21	0.408	0.01216224	3%	17.7%	0.49375	0.01463711	3%	19.6%	0.450875	0.06063441	13%	18.7%
1503 d0	207	positive	2.5665	2.6172	5/3/21	2.8742	2.8975	5/3/21	2.59185	0.03585031	1%	105.0%	2.88585	0.01647559	1%	105.3%	2.73885	0.20788939	8%	105.1%
1505 d0	208	positive	0.814	0.7716	5/6/21	0.9241	0.9189	5/6/21	0.7928	0.02998133	4%	34.4%	0.9215	0.00367696	0%	36.5%	0.85715	0.09100464	11%	35.5%
1506 d0	209	positive	1.161	1.1678	5/3/21	1.2052	1.2066	5/3/21	1.1644	0.00480833	0%	47.2%	1.2059	0.00098995	0%	44.0%	1.18515	0.02934493	2%	45.5%
1507 d0	210	positive	0.9082	0.9154	5/3/21	1.1142	1.0839	5/3/21	0.9118	0.00509117	1%	36.9%	1.09905	0.02142534	2%	40.1%	1.005425	0.13240574	13%	38.6%
1508 d0	211	positive	0.2354	0.2439	5/6/21	0.2906	0.3031	5/6/21	0.23965	0.00601041	3%	10.4%	0.29685	0.00883883	3%	11.8%	0.26825	0.04044651	15%	11.1%
0901 d0	212	positive	0.7645	0.7547	5/3/21	0.9127	0.9753	5/3/21	0.7596	0.00692965	1%	30.8%	0.944	0.04426488	5%	34.4%	0.8518	0.13039049	15%	32.7%
0902 d0	213	positive	1.4141	1.3277	5/3/21	1.6325	1.6805	5/3/21	1.3709	0.06109403	4%	55.5%	1.6565	0.03394113	2%	60.4%	1.5137	0.2019497	13%	58.1%
Squeaky	214	positive	0.4726	0.4641	5/3/21	0.6499	0.6615	5/3/21	0.46835	0.00601041	1%	19.0%	0.6557	0.00820244	1%	23.9%	0.562025	0.13247646	24%	21.6%
0302 d0	215	positive	1.3034	1.2113	5/6/21	1.4568	1.4854	5/6/21	1.25735	0.06512453	5%	54.6%	1.4711	0.02022325	1%	58.3%	1.364225	0.15114407	11%	56.5%
0703 d0	216	positive	1.8575	1.9637	5/3/21	2.2203	2.158	5/3/21	1.9106	0.07509474	4%	77.4%	2.18915	0.04405275	2%	79.9%	2.049875	0.19696459	10%	78.7%
0903 d0	217	positive	0.2574	0.2745	5/3/21	0.3957	0.3855	5/3/21	0.26595	0.01209153	5%	10.8%	0.3906	0.00721249	2%	14.2%	0.328275	0.08814086	27%	12.6%
1201 d0	218	positive	1.9876	2.0434	5/3/21	2.1841	2.094	5/3/21	2.0155	0.03945656	2%	81.7%	2.13905	0.06371032	3%	78.0%	2.077275	0.08736304	4%	79.7%
1510 d0	219	positive	2.1862	2.2923	5/3/21	2.5311	2.6694	5/3/21	2.23925	0.07502403	3%	90.7%	2.60025	0.09779287	4%	94.8%	2.41975	0.25526555	11%	92.9%
1511 d0	220	positive	1.1933	1.2561	5/3/21	1.5444	1.6545	5/3/21	1.2247	0.04440631	4%	49.6%	1.59945	0.07785246	5%	58.3%	1.412075	0.26498827	19%	54.2%
0704 d0	221	positive	1.7443	1.8905	5/3/21	2.2736	2.3997	5/3/21	1.8174	0.10337901	6%	73.6%	2.33665	0.08916617	4%	85.2%	2.077025	0.3671652	18%	79.7%
0303 d0	222	positive	1.8692	1.9224	5/3/21	2.2632	2.2117	5/3/21	1.8958	0.03761808	2%	76.8%	2.23745	0.036416	2%	81.6%	2.066625	0.24158303	12%	79.3%
1512 d0	223	positive	1.6833	1.7429	5/3/21	2.2766	2.2227	5/3/21	1.7131	0.04214356	2%	69.4%	2.24965	0.03811306	2%	82.1%	1.981375	0.37939814	19%	76.1%
Pos Control			2.5374	2.3992	5/3/21	2.7342	2.7489	5/3/21	2.4683	0.09772216	4%	100.0%	2.74155	0.01039447	0%	100.0%	2.604925	0.19321693	7%	100.0%
Neg Control			0.1424	0.1441	5/3/21	0.1718	0.1727	5/3/21	0.14325	0.00120208	1%	5.8%	0.17225	0.0006364	0%	6.3%	0.15775	0.0205061	13%	6.1%
0304 d0	224	positive	1.158	1.1385	5/4/21	0.905	0.9454	5/4/21	1.14825	0.01378858	1%	52.1%	0.9252	0.02856711	3%	46.5%	1.036725	0.15772017	15%	49.4%
0305 d0	225	positive	0.7284	0.7214	5/4/21	0.6317	0.5741	5/4/21	0.7249	0.00494975	1%	32.9%	0.6029	0.04072935	7%	30.3%	0.6639	0.08626703	13%	31.6%
1202 d0	226	positive	1.5525	1.6161	5/4/21	1.4018	1.4292	5/4/21	1.5843	0.04497199	3%	71.8%	1.4155	0.01937473	1%	71.1%	1.4999	0.11935962	8%	71.5%
1203 d0	227	positive	1.8536	1.8906	5/4/21	1.5557	1.5538	5/4/21	1.8721	0.02616295	1%	84.9%	1.55475	0.0013435	0%	78.1%	1.713425	0.22440034	13%	81.7%
Sac Stray	228	positive	1.2426	1.2124	5/4/21	1.0316	1.0129	5/4/21	1.2275	0.02135462	2%	55.7%	1.02225	0.0132229	1%	51.4%	1.124875	0.14513367	13%	53.6%
Potty Mouth	229	positive	1.7506	1.5932	5/4/21	1.5134	1.391	5/4/21	1.6719	0.11129861	7%	75.8%	1.4522	0.08654987	6%	73.0%	1.56205	0.15535136	10%	74.5%
1002	230	positive	0.8409	0.8421	5/4/21	0.6905	0.6923	5/4/21	0.8415	0.00084853	0%	38.2%	0.6914	0.00127279	0%	34.7%	0.76645	0.10613673	14%	36.5%
Bandit	231	positive	1.6261	1.4593	5/4/21	1.4618	1.4444	5/4/21	1.5427	0.11794541	8%	69.9%	1.4531	0.01230366	1%	73.0%	1.4979	0.06335677	4%	71.4%
Little Guy	232	positive	0.6696	0.6682	5/4/21	0.5482	0.534	5/4/21	0.6689	0.00098995	0%	30.3%	0.5411	0.01004092	2%	27.2%	0.605	0.09036825	15%	28.8%
0701 d0	233	positive	0.6791	0.6123	5/4/21	0.5403	0.5225	5/4/21	0.6457	0.04723473	7%	29.3%	0.5314	0.0125865	2%	26.7%	0.58855	0.08082231	14%	28.1%
0601 d0	234	positive	1.1169	1.1354	5/4/21	0.9933	0.9869	5/4/21	1.12615	0.01308148	1%	51.1%	0.9901	0.00452548	0%	49.7%	1.058125	0.09620188	9%	50.4%
1504-	301	negative	0.1102	0.1438	5/4/21	0.0925	0.0916	5/4/21	0.127	0.02375879	19%	5.8%	0.09205	0.0006364	1%	4.6%	0.109525	0.02471338	23%	5.2%
0702-	302	negative	0.0843	0.0785	5/4/21	0.0712	0.0751	5/4/21	0.0814	0.00410122	5%	3.7%	0.07315	0.00275772	4%	3.7%	0.077275	0.00583363	8%	3.7%
1509-	303	negative	0.1239	0.077	5/4/21	0.0733	0.0721	5/4/21	0.10045	0.03316331	33%	4.6%	0.0727	0.00084853	1%	3.7%	0.086575	0.01962221	23%	4.1%
1001-	304	negative	0.0792	0.0707	5/4/21	0.0659	0.0667	5/4/21	0.07495	0.00601041	8%	3.4%	0.0663	0.00056569	1%	3.3%	0.070625	0.00611647	9%	3.4%
1513-	305	negative	0.1172	0.1183	5/4/21	0.111	0.1043	5/4/21	0.11775	0.00077782	1%	5.3%	0.10765	0.00473762	4%	5.4%	0.1127	0.00714178	6%	5.4%
Fat Tony	306	negative	0.0938	0.0862	5/4/21	0.0871	0.0938	5/4/21	0.09	0.00537401	6%	4.1%	0.09045	0.00473762	5%	4.5%	0.090225	0.0003182	0%	4.3%
Squeak-	307	negative	0.0926	0.0865	5/4/21	0.086	0.0848	5/4/21	0.08955	0.00431335	5%	4.1%	0.0854	0.00084853	1%	4.3%	0.087475	0.00293449	3%	4.2%
Jamiraquoi	308	negative	0.0563	0.055	5/4/21	0.053	0.0539	5/4/21	0.05565	0.00091924	2%	2.5%	0.05345	0.0006364	1%	2.7%	0.05455	0.00155563	3%	2.6%
Prince	309	negative	0.0588	0.0568	5/4/21	0.0517	0.0518	5/4/21	0.0578	0.00141421	2%	2.6%	0.05175	7.0711E-05	0%	2.6%	0.054775	0.004278	8%	2.6%
0306 d0	310	negative	1.118	0.9837	5/4/21	1.0048	0.9433	5/4/21	1.05085	0.09496444	9%	47.6%	0.97405	0.04348707	4%	48.9%	1.01245	0.0543058	5%	48.3%
Pepper	235	positive	0.296	0.2882	5/4/21	0.2393	0.2262	5/4/21	0.2921	0.00551543	2%	13.2%	0.23275	0.0092631	4%	11.7%	0.262425	0.04196679	16%	12.5%
Aggie	401	positive	0.3277	0.3621	5/4/21	0.2973	0.3234	5/4/21	0.3449	0.02432447	7%	15.6%	0.31035	0.01845549	6%	15.6%	0.327625	0.02443054	7%	15.6%
BJ	402	positive	0.3855	0.367	5/4/21	0.2926	0.2859	5/4/21	0.37625	0.01308148	3%	17.1%	0.28925	0.00473762	2%	14.5%	0.33275	0.06151829	18%	15.9%
AJ	403	positive	0.7324	0.6552	5/4/21	0.5534	0.6023	5/4/21	0.6938	0.05458864	8%	31.5%	0.57785	0.03457752	6%	29.0%	0.635825	0.08198903	13%	30.3%
goose	404	positive	1.2331	1.186	5/4/21	1.0357	1.1243	5/4/21	1.20955	0.03330473	3%	54.8%	1.08	0.06264966	6%	54.3%	1.144775	0.09160568	8%	54.6%
Pos Control			2.1622	2.2489	5/4/21	1.9409	2.0402	5/4/21	2.20555	0.06130616	3%	100.0%	1.99055	0.0702157	4%	100.0%	2.09805	0.15202796	7%	100.0%
Neg Control			0.1183	0.1318	5/4/21	0.1088	0.1076	5/4/21	0.12505	0.00954594	8%	5.7%	0.1082	0.00084853	1%	5.4%	0.116625	0.01191475	10%	5.6%
95	101	negative	0.0583	0.06	5/5/21	0.0553	0.0515	5/5/21	0.05915	0.00120208	2%	5.3%	0.0534	0.00268701	5%	5.2%	0.056275	0.00406586	7%	5.2%
109	102	negative	0.0517	0.0523	5/5/21	0.0552	0.0515	5/5/21	0.052	0.00042426	1%	4.6%	0.05335	0.0026163	5%	5.1%	0.052675	0.00095459	2%	4.9%
452	103	negative	0.0517	0.0525	5/5/21	0.0499	0.0555	5/5/21	0.0521	0.00056569	1%	4.6%	0.0527	0.0039598	8%	5.1%	0.0524	0.00042426	1%	4.8%
575	104	negative	0.0771	0.07	5/5/21	0.0722	0.0647	5/5/21	0.07355	0.00502046	7%	6.5%	0.06845	0.0053033	8%	6.6%	0.071	0.00360624	5%	6.6%
559	105	negative	0.0658	0.0691	5/5/21	0.0677	0.0677	5/5/21	0.06745	0.00233345	3%	6.0%	0.0677	0	0%	6.5%	0.067575	0.00017678	0%	6.3%
613	106	negative	0.0641	0.0668	5/5/21	0.0602	0.06	5/5/21	0.06545	0.00190919	3%	5.8%	0.0601	0.00014142	0%	5.8%	0.062775	0.00378302	6%	5.8%
664	107	negative	0.1005	0.0986	5/5/21	0.0909	0.0859	5/5/21	0.09955	0.0013435	1%	8.8%	0.0884	0.00353553	4%	8.5%	0.093975	0.00788424	8%	8.7%
0800 d0	108	negative	0.1405	0.1263	5/6/21	0.1545	0.1537	5/6/21	0.1334	0.01004092	8%	5.8%	0.1541	0.00056569	0%	6.1%	0.14375	0.01463711	10%	6.0%
842	109	negative	0.0761	0.0676	5/5/21	0.0657	0.0689	5/5/21	0.07185	0.00601041	8%	6.4%	0.0673	0.00226274	3%	6.5%	0.069575	0.00321734	5%	6.4%
0907 d0	110	negative	0.0971	0.0956	5/6/21	0.1157	0.1096	5/6/21	0.09635	0.00106066	1%	4.2%	0.11265	0.00431335	4%	4.5%	0.1045	0.01152584	11%	4.3%
923	111	negative	0.0556	0.0591	5/5/21	0.0583	0.0513	5/5/21	0.05735	0.00247487	4%	5.1%	0.0548	0.00494975	9%	5.3%	0.056075	0.00180312	3%	5.2%
1024 d0	112	negative	0.1111	0.1161	5/6/21	0.1216	0.1311	5/6/21	0.1136	0.00353553	3%	4.9%	0.12635	0.00671751	5%	5.0%	0.119975	0.00901561	8%	5.0%
1130 d0	113	negative	0.0966	0.1038	5/6/21	0.0996	0.1042	5/6/21	0.1002	0.00509117	5%	4.3%	0.1019	0.00325269	3%	4.0%	0.10105	0.00120208	1%	4.2%
1440	114	negative	0.0621	0.0603	5/5/21	0.0556	0.061	5/5/21	0.0612	0.00127279	2%	5.4%	0.0583	0.00381838	7%	5.6%	0.05975	0.00205061	3%	5.5%
1474	115	negative	0.0696	0.061	5/5/21	0.0694	0.0671	5/5/21	0.0653	0.00608112	9%	5.8%	0.06825	0.00162635	2%	6.6%	0.066775	0.00208597	3%	6.2%
9491	116	negative	0.058	0.0541	5/5/21	0.0566	0.054	5/5/21	0.05605	0.00275772	5%	5.0%	0.0553	0.00183848	3%	5.3%	0.055675	0.00053033	1%	5.2%
9687	117	negative	0.0602	0.0629	5/5/21	0.0599	0.0621	5/5/21	0.06155	0.00190919	3%	5.5%	0.061	0.00155563	3%	5.9%	0.061275	0.00038891	1%	5.7%
9857	118	negative	0.0554	0.0537	5/5/21	0.0559	0.0567	5/5/21	0.05455	0.00120208	2%	4.8%	0.0563	0.00056569	1%	5.4%	0.055425	0.00123744	2%	5.1%
zag1 d0	119	negative	0.0751	0.0791	5/5/21	0.0754	0.0752	5/5/21	0.0771	0.00282843	4%	6.9%	0.0753	0.00014142	0%	7.3%	0.0762	0.00127279	2%	7.1%
mdo6 do	120	negative	0.0739	0.075	5/5/21	0.0722	0.0744	5/5/21	0.07445	0.00077782	1%	6.6%	0.0733	0.00155563	2%	7.1%	0.073875	0.00081317	1%	6.8%
zae1 d0	121	negative	0.0723	0.0759	5/5/21	0.0711	0.0716	5/5/21	0.0741	0.00254558	3%	6.6%	0.07135	0.00035355	0%	6.9%	0.072725	0.00194454	3%	6.7%
meb3 d0	122	negative	0.0729	0.0772	5/5/21	0.0751	0.0724	5/5/21	0.07505	0.00304056	4%	6.7%	0.07375	0.00190919	3%	7.1%	0.0744	0.00091924	1%	6.9%
0307 d0	236	positive	0.9163	0.8662	5/5/21	0.845	0.8181	5/5/21	0.89125	0.03542605	4%	79.2%	0.83155	0.01902117	2%	80.3%	0.8614	0.04221427	5%	79.7%
Charm	237	positive	0.159	0.1604	5/5/21	0.1463	0.1538	5/5/21	0.1597	0.00098995	1%	14.2%	0.15005	0.0053033	4%	14.5%	0.154875	0.00682358	4%	14.3%
Pos Control			1.1503	1.1005	5/5/21	1.0649	1.0074	5/5/21	1.1254	0.03521392	3%	100.0%	1.03615	0.04065864	4%	100.0%	1.080775	0.06310928	6%	100.0%
